# A case displaying the importance of JAK1 and JAK2 gene transcription in antifungal defense against coccidioidomycosis

**DOI:** 10.3389/fmed.2025.1643068

**Published:** 2025-10-14

**Authors:** Jennifer K. Priessnitz, Taylor Colore, Nelson Nicolasora

**Affiliations:** ^1^Division of Internal Medicine, University of Arizona College of Medicine - Phoenix, Phoenix, AZ, United States; ^2^Division of Internal Medicine, Macon & Joan Brock Virginia Health Sciences at Old Dominion University, Norfolk, VA, United States; ^3^St. George's University School of Medicine, Grenada, West Indies; ^4^Division of Infectious Disease, University of Arizona - Phoenix, Phoenix, AZ, United States

**Keywords:** JAK1 and JAK2 inhibitors, ruxolitinib, coccidioidomycosis, coccidioidomycosis/immunology, JAK-STAT signaling pathway, antifungal immunity

## Abstract

This case report explores the consequences of ruxolitinib via inhibition janus kinase 1 (JAK1) and JAK2 pathways in the context of fungal defense in a patient diagnosed with pulmonary coccidioidomycosis during ruxolitinib therapy for polycythemia vera. The patient experienced a relapse of pulmonary coccidioidomycosis after antifungal treatment was discontinued while continuing ruxolitinib use. This case illustrates the heightened risk of discontinuing antifungal therapy in endemic regions, emphasizing the critical need for continued monitoring. Furthermore, this case underscores the vital role of the JAK1 and JAK2 signaling cascade, particularly the interferon-gamma (INF-γ)-JAK1 and JAK2-signal transducer and activator of transcription 1 (STAT) axis, in antifungal defense. Recent studies have revealed that the loss of function in JAK1 (but not JAK2), leads to impaired macrophage activation and reduced T-helper 1 (Th1) cell responses, thereby compromising the body's ability to fight off dimorphic fungi, such as *Coccidioides*. Other proposed fungal immune mechanisms in the JAK-STAT pathway are discussed. Clinicians tailoring JAK inhibitor treatment options for patients must be aware of the INF-γ-JAK1-STAT pathway's pivotal role in antifungal defense.

## Introduction

The Janus kinase-signal transducer and activator of transcription (JAK-STAT) pathway is a well-known cornerstone in immune regulation, orchestrating a variety of cellular responses via cytokine signaling. Inhibition of JAK1 and JAK2 can compromise the immune system's ability to defend against infections. This case report explores the consequences of inhibiting JAK1 and JAK2 via ruxolitinib, particularly in the context of fungal defense in a patient diagnosed with pulmonary coccidioidomycosis.

Ruxolitinib, a selective JAK1 and JAK2 inhibitor, is frequently used to treat polycythemia vera (PV), myelofibrosis, and graft-vs.-host disease (GVHD). While effective for controlling disease progression, its immunosuppressive effects can increase susceptibility to infections. Notably, fungal infections were not prominently featured in early clinical trials (1, 2). However, more recent studies and case reports have documented occurrences of invasive fungal infections in patients treated with ruxolitinib (3, 4).

We present a case of a 60-year-old male residing in Arizona who developed pulmonary coccidioidomycosis during ruxolitinib therapy. Following treatment with itraconazole, symptoms and radiologic imaging improved, and antifungal therapy was discontinued after 18 months of treatment. A year later, the patient redeveloped pulmonary coccidioidomycosis with continued ruxolitinib treatment. This case illustrates the potential consequences of discontinuing antifungal therapy, raising concerns about prophylaxis, and underscoring the critical role of the JAK1 and JAK2 signaling cascade, particularly the interferon gamma (IFN-γ)-JAK1 and JAK2-STAT1 axis, in antifungal defense (3, 5–7). Informed consent was affirmed and obtained by the patient, with data privacy safeguards.

## Case presentation

**Figure 1:**
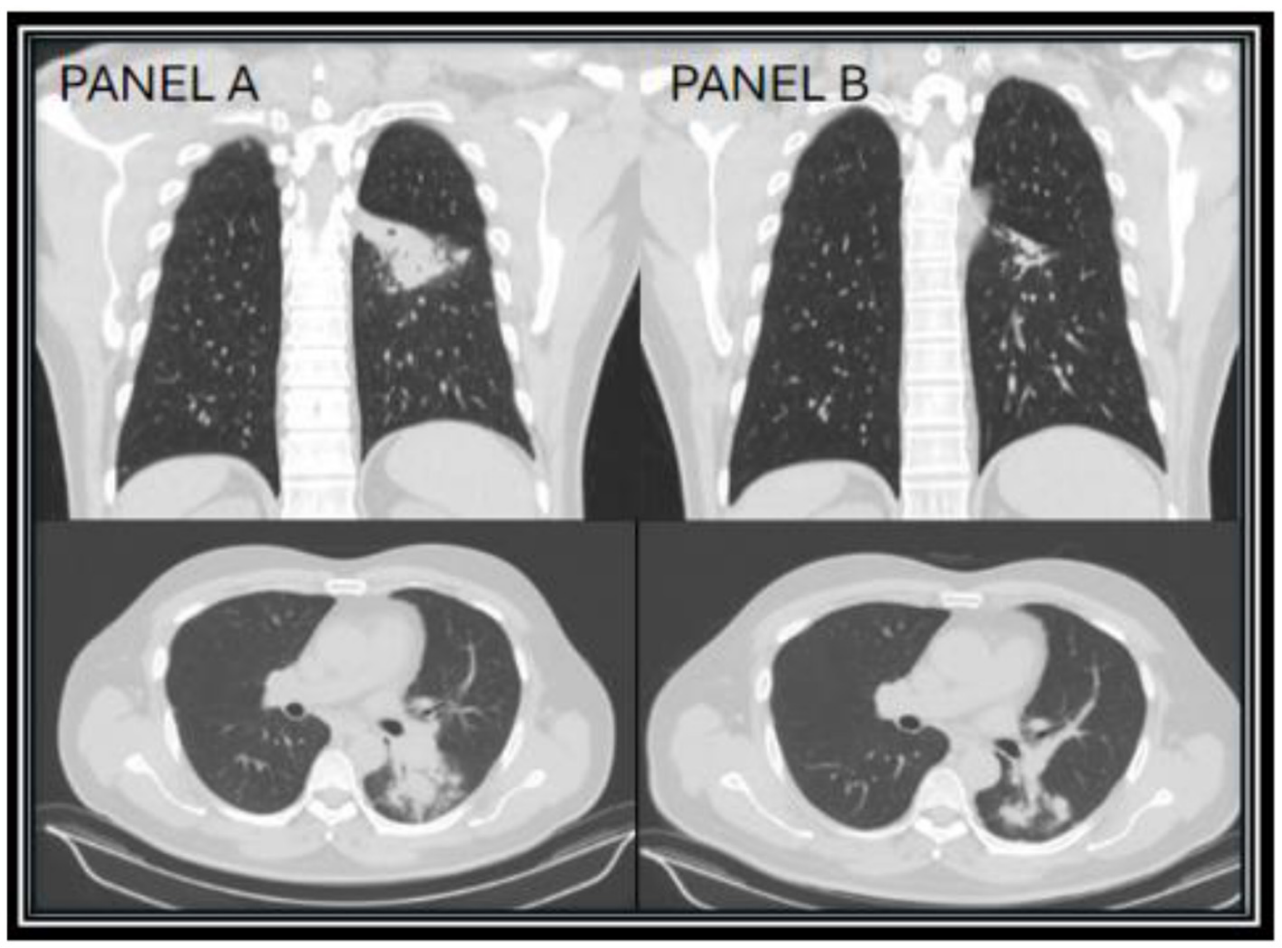
CT chest images taken 1 month after diagnosis (year zero) of Coccidioides infection showing the superior left lower lobe infiltrate **(A)**, and repeat CT chest taken 17 months on itraconazole showing improvement of the superior left lower lobe infiltrate **(B)**.

A 60-year-old male residing in Arizona with a history of PV on ruxolitinib therapy presented with a four-week history of productive cough with whitish sputum and intermittent fever. A chest computed tomography (CT) scan demonstrated a left lower lobe consolidation accompanied by satellite nodules and mediastinal lymphadenopathy (Figure 1A). He was initially treated with oseltamivir and levofloxacin by his primary care physician, without clinical improvement. Serologic testing returned positive for both *Coccidioides* IgM and IgG by immunoassay, with a complement fixation (CF) titer of 1:4. His diagnosis of pulmonary coccidioidomycosis was confirmed via a fungal sputum culture, and antifungal therapy with fluconazole was initiated; however, due to persistent symptoms after 2 weeks, he was transitioned to itraconazole. Itraconazole, a strong CYP3A4 inhibitor, is known to increase ruxolitinib blood levels. Given the potential for drug–drug interaction between itraconazole and ruxolitinib, the patient's hematologist reduced the patient's ruxolitinib dose by 50% from per oral (PO) 10 mg twice daily (BID) to PO 5 mg BID, with monthly monitoring of the patient's complete blood count, which remained within normal limits. The patient's serum itraconazole and hydroxyitraconazole levels remained within therapeutic range (>1 mcg/mL) throughout the patient's clinical course while on the antifungal, which was measured every 6 months.

After discussions with infectious disease and hematology regarding continued treatment of ruxolitinib in the setting of an active infection, the patient decided to continue ruxolitinib in conjunction with itraconazole, due to the patient's excellent disease control of his PV on ruxolitinib. On repeat chest CT done at 17 months of treatment, the patient's left lower lobe consolidation showed near resolution, with small residual fibrotic changes (Figure 1B). At the patient's follow up visit, he endorsed complete resolution of symptoms, with clinical improvement over 18 months. Due to presumed disease control, no elevation in CF titers (1:4), and proper IDSA recommended treatment length in immunocompromised patients, itraconazole was discontinued and the patient was counseled to call their provider with any relapse of symptoms (8). Additionally, 2 months later, the ruxolitinib dose was increased to PO 10 mg BID.

**Figure 2:**
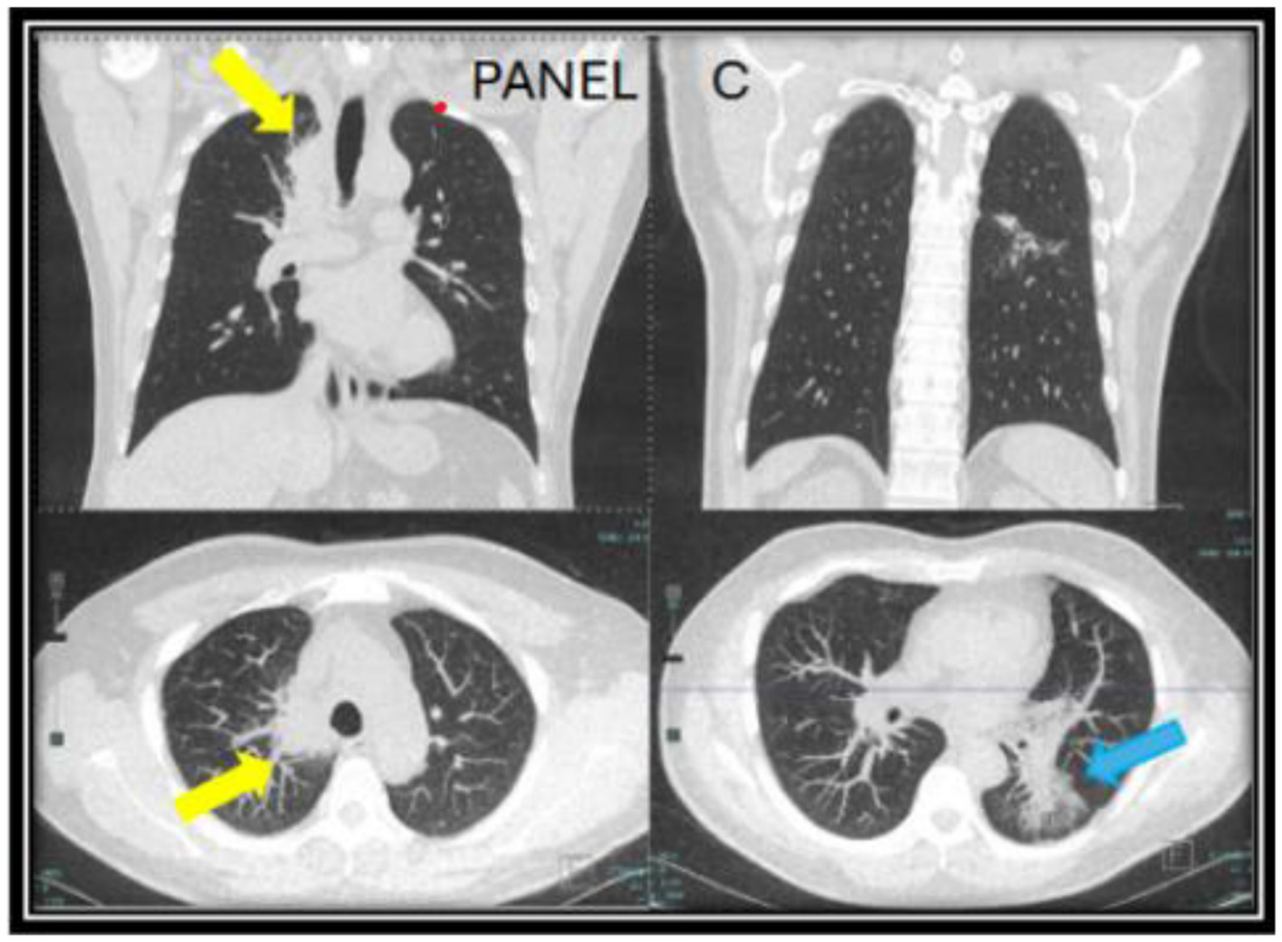
CT chest images taken a year after of being off of itraconazole (year two) when the patient developed a new persistent cough associated with a rise in Coccidioides complement fixation titers. A new mass-like consolidation of the medial right lung apex measuring 5.1 × 2.9 × 0.8 cm **(yellow arrow)** was noted. The superior segment of the left lower lobe continued to improve **(blue arrow)**. The nodular consolidation has decreased, and mild scarring and bronchiectasis now persisted in this area **(C)**.

**Figure 3:**
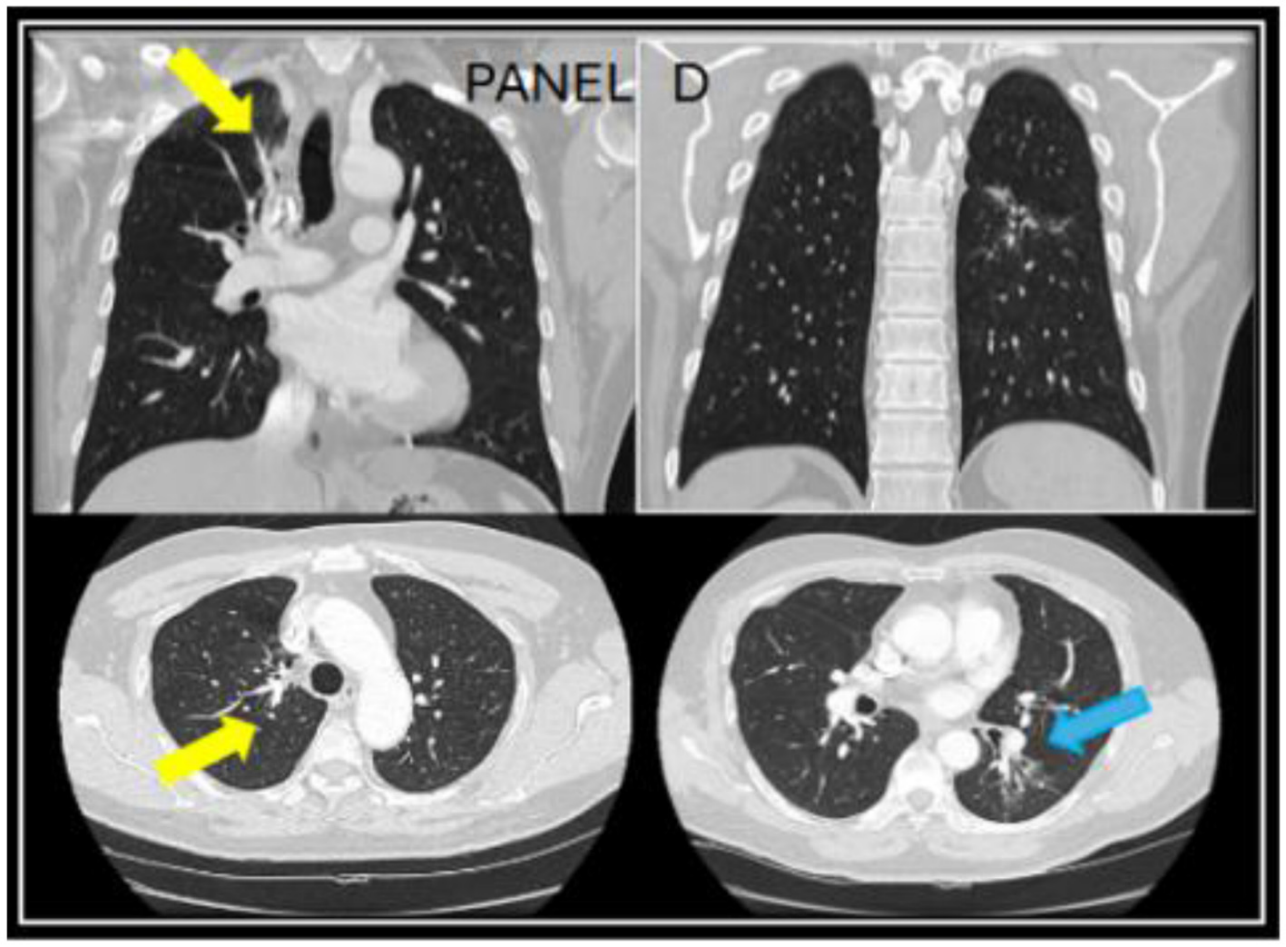
A follow up CT chest done at year four while being on long-term itraconazole showed resolution of the right upper lung infiltrate **(yellow arrows)** and residual reticulated bronchiectasis in the left lower lobe [**blue arrow** in **(D)**].

However, the patient expressed concerns about a new onset dry cough a year later. He was urged to present to the clinic for a clinical workup, but he did not follow up for 6 months. When he presented to the clinic, he continued to endorse persistent symptoms of dry cough and shortness of breath. A CT chest revealed a new right upper lobe infiltrate, and the patient's *Coccidioides* CF titer had spiked to 1:64 (Figure 2). Recognizing this as a relapse of pulmonary coccidioidomycosis, the patient was promptly restarted on itraconazole, which resulted in dramatic resolution of symptoms within 2 weeks. With initiation of itraconazole, the patient's ruxolitinib dose was again reduced to PO 5 mg BID. Follow-up testing showed a steady decline in CF titers to 1:4, confirming the effectiveness of continued antifungal therapy. Repeat CT imaging revealed resolution of right upper lobe infiltrate after 2 years of itraconazole use (Figure 3). At the time of publication of this paper, the patient has continued to maintain effective disease control on itraconazole and ruxolitinib and will continue with indefinite antifungal treatment.

**Figure 4:**
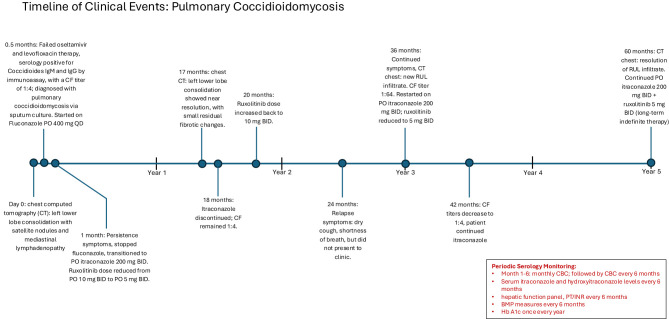
A timeline of the patient's clinical course.

Of note, the patient had no other concomitant risks for immunosuppression during his clinical course. He had no episodes of absolute lymphopenia, leukopenia or neutropenia, which was monitored every 6 months. Hepatic function was monitored via hepatic panel, and hepatic synthetic function (albumin, prothrombin time, international normalized ratio) which remained within normal limits, measured every 6 months. Renal function was monitored via basic metabolic panel which remained within normal limits, measured every 6 months. The patient did not have diabetes, which was monitored via hemoglobin A1c yearly and fasting blood sugar, and was not on any other immunosuppressant medications. Patient's clinical course is depicted in a timeline in Figure 4.

## Discussion

The JAK-STAT signaling pathway plays a pivotal role in immune regulation, particularly in modulating responses to infections. In the RESPONSE phase 3 clinical trial for ruxolitinib, it was reported that infections occurred in 41.8% of patients taking ruxolitinib vs. 36.9% in the standard therapy group for patients with PV (1). The most frequently associated infections in patients taking ruxolitinib include urinary tract infections (4.7–24.6%), pneumonia (5.3–13.1%), herpes zoster (1.9–11.5%), and sepsis (1.3–7.9%) (9). Other infections, such as tuberculosis, toxoplasmosis, progressive multifocal leukoencephalopathy, and hepatitis B reactivation, have also been reported (10, 11). In the RESPONSE-1 and RESPONSE-2 clinical trials, opportunistic fungal infections were not significantly different between patients taking ruxolitinib and those in the control group (2, 12). In a systematic review conducted by Lussana et al., patients taking ruxolitinib were found to have an increased risk of herpes zoster infection but did not report an increased risk of fungal infections (10). Nonetheless, numerous cases in literature identify patients on ruxolitinib with disseminated or severe opportunistic fungal infections, such as cryptococcal pneumonia, *Pneumocystis jirovecii*, and Rhizomucor pulmonary infections (4, 13–16). Chiu et al. investigated a systematic review identifying 28 patients with PV or myelofibrosis treated with ruxolitinib who experienced invasive fungal infections. The most common pathogens were *Cryptococcus* (46%), *Coccidioides* (11%), and *Pneumocystis jirovecii* (11%). In most cases, ruxolitinib was discontinued permanently (76%) or put on hold (18%). Additionally, no patient had received antifungals prior to invasive fungal infection diagnosis (3). These cases underscore the increased susceptibility to fungal pathogens in patients on JAK1 and JAK2 inhibitors (Table 1). Patient diagnoses of mycotic infections in these various studies used serologic testing and sensitivities based off of the IDSA guidelines CDC reports, and large cohort studies (Appendix Tables A1, A2).

**Table 1:** Reported endemic mycoses associated with JAK1/JAK2 inhibitors and clinical presentations.

**Mycosis**	**JAK inhibitor involved**	**Reported clinical presentation**	**Relevant references**
Coccidioidomycosis	Ruxolitinib (JAK1/JAK2)	8 cases: 4 pulmonary coccidioidomycosis, 3 multisite disseminated to skin, bone, liver, and spleen, 1 skin coccidioidomycosis	(4)
	STAT3 GOF treated with ruxolitinib (JAK1/JAK2)	Disseminated coccidiomycosis	(40)
	STAT1 GOF treated with ruxolitinib (JAK1/JAK2)	Disseminated coccidiomycosis to L medial rectus muscle	(44)
	Ruxolitinib (JAK1/JAK2)	1 patient: unspecified clinical presentation	(16)
	Ruxolitinib (JAK1/JAK2)	Pulmonary coccidiomycosis	Current Case Report (Priessnitz et al.)
	Upadacitinib (JAK1)	unspecified	(30, 31)
Histoplasmosis	Ruxolitinib (JAK1/JAK2)	Disseminated histoplasmosis with fevers, pulmonary nodules	(3)
		1 patient: unspecified clinical presentation	(16)
Cryptococcosis	Ruxolitinib (JAK1/JAK2)	Disseminated cryptococcal pneumonia	(3)
	STAT3 GOF treated with ruxolitinib (JAK1/JAK2)	1 patient with Cryptococcal pneumonia	(40)
	Ruxolitinib (JAK1/JAK2)	1 patient: unspecified clinical presentation	(16)
	upadacitinib (JAK1)	Unspecified	(26)
*Pneumocystis jirovecii* pneumonia	Ruxolitinib (JAK1/JAK2), Upadacitinib (JAK1)	Severe pneumonia requiring hospitalization	(3)
	STAT3 GOF treated with ruxolitinib (JAK1/JAK2)	2 patients: Severe pneumonia requiring hospitalization	(40)
	Ruxolitinib (JAK1/JAK2)	3 patients: unspecified clinical presentation	(16)
	Upadacitinib (JAK1)	Unspecified	(26)
	Upadacitinib (JAK1)	Unspecified	(30, 31)
Aspergillosis	Ruxolitinib (JAK1/JAK2)	5 patients	(16)
	upadacitinib (JAK1)	Unspecified	(30, 31)
Candidiasis	Ruxolitinib (JAK1/JAK2)	1 patient	(14)
	Ruxolitinib (JAK1/JAK2)	6 patients with Candidiasis: unspecified clinical presentation	(16)
dermatophytosis	STAT1 GOF treated with ruxolitinib (JAK1/JAK2)	bilateral feet, acutely worsened on treatment and resistant to all antifungal medications	(44)

When reviewing the literature for patients on JAK1 and JAK2 inhibitors that developed coccidioidomycosis, a case series by Kusne et al. documents eight cases of coccidioidomycosis in patients receiving ruxolitinib in endemic regions of the San Joaquin Valley of California and Arizona. Three of these patients had primary pulmonary coccidioidomycosis prior to ruxolitinib, with treatment with antifungals that were continued after initiation of ruxolitinib, resulting in no further disease. In the other five cases, coccidioidomycosis developed after the initiation of ruxolitinib. All five patients presented with symptoms, including bilateral pulmonary nodules or extrathoracic dissemination to the skin, bones, liver, and spleen. In all five cases, appropriate antifungal treatment was started, and ruxolitinib was discontinued indefinitely. One of these patients had a history of coccidioidomycosis, who was not on immunosuppression, and had been successfully treated for 1 year. Five years after antifungal treatment, the patient started ruxolitinib therapy with negative *Coccidioides* CF titers. However, two years into ruxolitinib treatment, the patient developed multisite disseminated coccidioidomycosis, necessitating the discontinuation of ruxolitinib and initiation of antifungal therapy (4). Similarly, both the patient described by Kusne as well as the patient in our case developed a relapse of pulmonary *Coccidioides* infection while on ruxolitinib. However, unlike other cases, our patient successfully continued ruxolitinib during both the initial infection and the relapse, achieving resolution with antifungal therapy. The relapse following antifungal discontinuation highlights the risks of stopping therapy in endemic regions. In the literature, there is ongoing debate as to whether the increased risk of fungal infections in patients taking ruxolitinib is attributable to the underlying hematologic disease (PV or myelofibrosis) or the immunosuppressive effects of the drug itself. Our case supports the latter, as the patient's PV remained largely stable throughout the treatment course, while the relapse of pulmonary coccidioidomycosis occurred following the cessation of antifungal therapy (3).

The current consensus for JAK1 and JAK2 inhibitors does not support antifungal prophylaxis (8). Clinicians should be made aware of the heightened risk of relapse of fungal infections in patients on JAK1 and JAK2 inhibitors. Specifically, our case highlights the critical importance of regularly screening for fungal infections, particularly in patients residing in endemic regions. This case suggests that there may be a role for continued long-term antifungal prophylaxis, particularly in high-risk patients who have experienced a relapse while on JAK inhibitors. However, the decision to implement prophylaxis must carefully balance the benefits of infection prevention against the potential risks, including drug interactions, azole resistance, and medication-induced organ damage.

### JAK-STAT proposed immune regulation of dimorphic fungi

**Figure 5:**
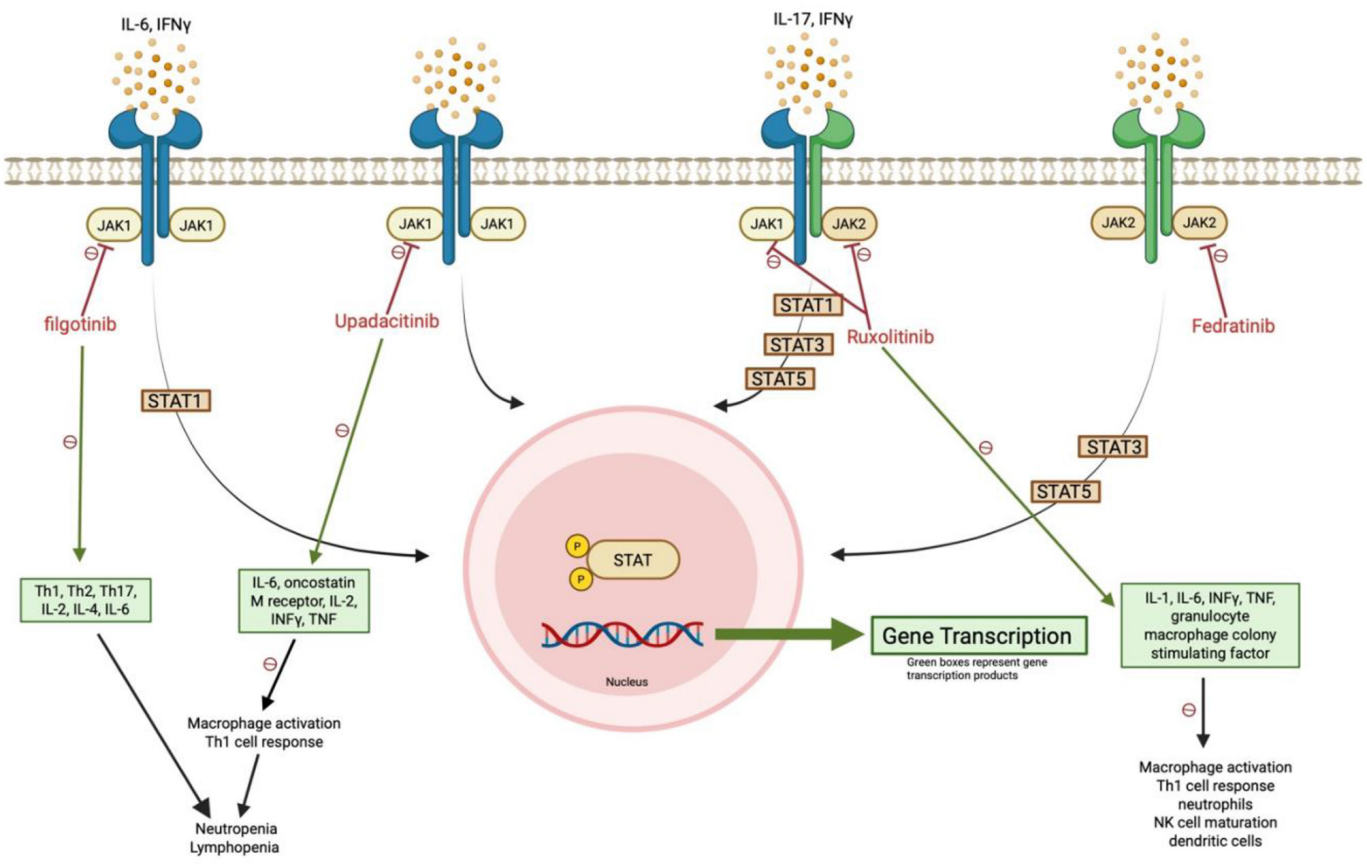
JAK1/JAK2 inhibition disrupts antifungal immune signaling. Ruxolitinib and other selective JAK inhibitors impair STAT phosphorylation and downstream gene transcription, reducing Th1 responses, macrophage activation, and innate immune cell function. These effects, particularly from JAK1 blockade, may compromise defense against dimorphic fungi such as *Coccidioides*. **Green boxes** indicate affected gene transcription products.

**Figure 6:**
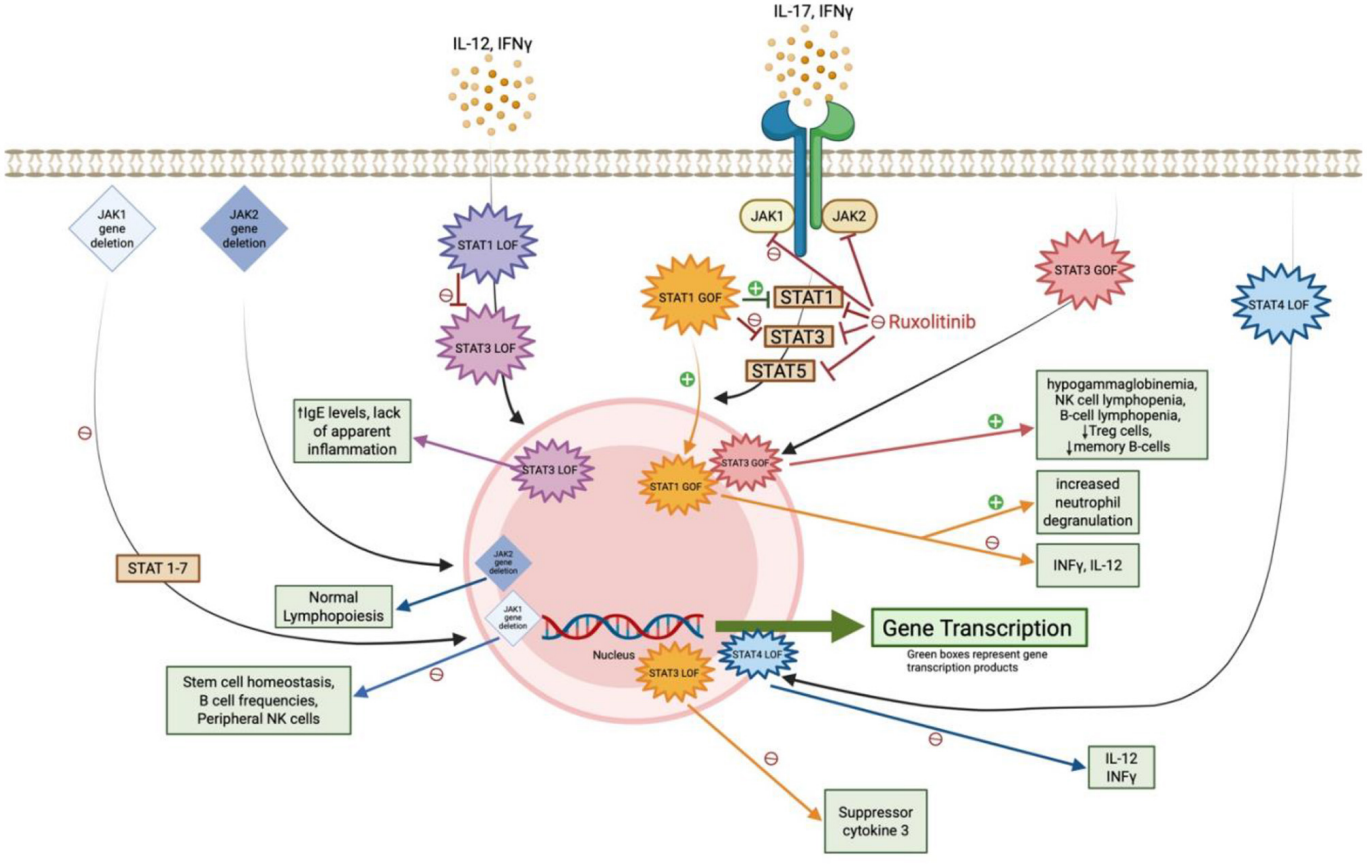
Impact of JAK-STAT gene mutations and ruxolitinib on antifungal immunity. This diagram illustrates how JAK1/JAK2 gene deletions and STAT gain- or loss-of-function (GOF/LOF) mutations influence downstream immune signaling. **Green boxes** denote key transcription products involved in antifungal defense; disruptions in these pathways contribute to increased susceptibility to endemic mycoses.

Our case suggests that an increased risk for dimorphic fungal infections, such as *Coccidioides*, in endemic areas is associated with ruxolitinib-mediated inhibition of the JAK1 and JAK2 pathways, compared to normal exposure risk alone. This section will review proposed JAK-STAT immune mechanisms that may contribute to heightened risk of infections with dimorphic fungi with Figures 5, 6 also highlighting the various pathways.

Ruxolitinib's mechanism of action involves inhibition of JAK1 and JAK2, which prevents the tyrosine phosphorylation of the various seven STAT proteins. This blockade disrupts their transport to the nucleus, thereby impeding downstream gene transcription of cytokine proliferation (3, 6, 17–19). JAK1 and JAK2 signaling, particularly through INF-γ-JAK-STAT pathways, is essential for activating macrophages, dendritic cells, and T-helper 1 (Th1) cell responses, which are crucial for controlling fungal pathogens like *Coccidioides* (3, 5–7). This pathway has downstream effects on cytokines such as IL-1, IL-6, INF-γ, tumor necrosis factor-alpha (TNF-α), and granulocyte-macrophage colony-stimulating factor, all of which recruit neutrophils and mononuclear phagocytes (3, 17, 19, 20). Additionally, recent studies suggest that ruxolitinib may impair the maturation and function of natural killer (NK) cells and dendritic cells (17). Animal studies involving loss of function (LOF) or gain of function (GOF) gene mutations targeting the JAK or STAT pathways have identified specific fungal immunity signaling cascades critical for immune cell activation and function. However, the precise mechanism by which the JAK-STAT pathway controls *Coccidioides* remains unclear. A study of primary immune deficiency cases involving disseminated coccidioidomycosis found two patients had STAT3 LOF mutations, one had IFN-γ receptor-1 deficiency, three had IL-12 receptor LOF mutations, and two had STAT1 GOF mutations. These findings suggest that the IL-12/IFN-γ axis and STAT3-mediated immunity play a role in protection against coccidiomycosis (21).

### JAK1 inhibition's role on fungal immunity

Looking deeper into the JAK inhibitor pathways, recent studies have suggested that the loss of function in JAK1, in particular, leads to impaired macrophage activation and reduced Th1 cell responses, thereby compromising the body's ability to combat dimorphic fungi, such as *Coccidioides* (17). In contrast, selective inhibition of JAK2 alone does not appear to have the same profound impact on fungal immunity. This concept is highlighted by a case of a 59-year-old woman with myelofibrosis who was treated with ruxolitinib and developed disseminated histoplasmosis. The infection improved after discontinuation of ruxolitinib and proper antifungal treatment. As fedratinib, a selective JAK2 inhibitor is approved for use in myelofibrosis (but not for PV as in our case), the patient was switched to fedratinib without experiencing a relapse of histoplasmosis or other fungal infections (3). While selective JAK1 or JAK2 inhibitors are not used for treatment of PV, this case underscores the importance of these pathways in antifungal immunity. Further studies in mouse models support this notion. Inducible deletion of JAK1, which blocks phosphorylation of all STAT proteins, significantly disrupts stem cell homeostasis and reduces B cell frequencies (22, 23). Similarly, NK cell-specific deletion of JAK1 severely reduces peripheral NK cell populations (24). In contrast, inducible deletion of JAK2 does not impact lymphopoiesis, and NK cell-specific JAK2 deficiency does not interfere with NK-cell homeostasis (22, 24, 25).

Moreover, newly approved selective JAK1 inhibitors, such as upadacitinib and filgotinib, drug-reported safety profile discloses an increased risk for invasive fungal infections, such as cryptococcosis and pneumocystis (upadacitinib only) and endemic mycosis infections, as well as an increased risk of neutropenia and lymphopenia (26, 27). Filgotinib inhibits Th1, Th2, and Th17 differentiation, as well as cytokines IL-2, IL-4, and IL-6, which play critical roles in immunity (28, 29). Robust Th1 and Th17 immune response have been shown to be crucial for combating *Coccidioides*, and inhibition of both have been associated with chronic and disseminated coccidioidomycosis (17, 28). Upadacitinib inhibits cytokines such as IL-6, oncostatin M receptor, IL-2, IFNγ, and TNF and is involved in immune cell migration and inflammatory responses (28). In a review article, an increased risk of pneumocystis carinii pneumonia was observed in 6.1% of patients treated with upadacitinib 30 mg BID over 84 weeks. Endemic coccidioidomycosis was reported in 0.2% of patients receiving upadacitinib 6–12 mg BD over 72 weeks, and aspergillosis was found in 0.15% of patients receiving upadacitinib 15 mg OD over 48 weeks. These findings raise important questions regarding the immunological behavior of selective JAK1 inhibition (30, 31). Notably, the selective JAK1 inhibitor, itacitinib, was discontinued after phase 3 clinical trials due to its ineffectiveness in treatment of GVHD, and there are no current data on the risks of fungal infections with this drug (32). This suggests that the JAK1-STAT pathway plays a pivotal role in defense against fungal infections.

### STAT gene mutation's role on fungal immunity

In a comprehensive review of JAK-STAT defects and immune dysregulation conducted by Chaimowitz et al., an increased risk of invasive fungal infections was noted due to various immune mechanisms. In comparison to our case, potential immune dysregulation resulting from the inhibition of JAK-STAT pathways will be discussed below.

### STAT3 LOF

Inhibition of JAK1 prevents the phosphorylation of STAT3, leading to reductions in STAT3 downstream gene transcription. This phenotype can resemble that identified patients with an autosomal dominant STAT3 LOF mutation, also called hyper-IgE syndrome (33). These patients present with elevated IgE levels (>2,000 U/mL), recurrent pneumonia and recurrent staphylococcal skin abscesses with a lack of apparent inflammation (34). They often develop bronchiectasis and pneumatoceles and are at a higher risk for fungal infections such as *Aspergillus*, requiring prophylaxis with itraconazole. It is also noted that endemic mycoses frequently cause disseminated disease including histoplasmosis, *Coccidioides*, and *Cryptococcus*, requiring prophylaxis for high-risk exposure in all patients identified with a STAT3 LOF gene mutation (35, 36). Alternatively, discussed earlier in this case report, Chiu et al. described a patient who developed disseminated histoplasmosis while on the selective JAK1 and JAK2 inhibitor, ruxolitinib. The histoplasmosis is completely resolved with a proper antifungal treatment course and switching treatment to the selective JAK2 inhibitor fedratinib (3). Fedratinib's immune mechanism is thought to inhibit the phosphorylation of STAT3 and STAT5, which could correlate with a STAT3 LOF gene mutation, challenging the idea that endemic mycoses are associated with STAT3 LOF mutations (28).

### STAT3 GOF

Although the JAK1 pathway is not correlated with increased phosphorylation of STAT3, patients with a STAT3 GOF mutation were susceptible to severe infections. These include viral infections, nontuberculous mycobacterial infections, fungal infections, and opportunistic infections, often accompanied by presentations of hypogammaglobulinemia, NK cell lymphopenia, T cell lymphopenia, B-cell lymphopenia, decreased in regulatory T cells and decreased memory B cells (36, 37). It has been noted that patients with T cell lymphopenia are at higher risk of viral and/or fungal infections (38, 39). In a review by Forbes et al., it was reported that 16 patients with STAT3 GOF mutations were treated with ruxolitinib to treat their immune defects. Interestingly, one patient developed disseminated coccidioidomycosis while being treated on ruxolitinib. The dose of ruxolitinib was reduced, although the patient ultimately died 6 months later due to the progression of disseminated coccidioidomycosis (40). This highlights that STAT3 GOF mutations may also contribute to dimorphic fungal immunity pathways.

### STAT1 GOF

Although inhibition of the JAK1 pathway is not correlated with increased phosphorylation of STAT1, novel research reported that in patients with STAT1 GOF gene mutations, despite treatment with ruxolitinib for improvement of their chronic mucocutaneous candidiasis (CMC), patients still had an increased risk for disseminated endemic mycoses (41, 42). Patients with STAT1 GOF mutations present with CMC due to impaired IL-17 differentiation, leading to a dysregulation in STAT1/STAT3 signaling. This results in increased STAT1 gene transcription and a reduction in the expression of STAT3 dependent genes, such as suppressor of cytokine 3 (SOCS3), which contributes to reduced antifungal immunity (36, 43–45). Selective JAK1 and JAK2 inhibition with ruxolitinib has been shown to improve the dysregulation of increased phosphorylation of STAT1, leading to enhanced activation of T cells and B cells, leading to increased activation of CD45, CD52, and CD99, with partial restoration of NK cell cytotoxic function. These effects are highly effective in treating severe CMC and restoring the patient's immune system (36, 45–48).

However, Zimmerman et al. reported that two of six patients with STAT1 GOF mutations, who were treated with ruxolitinib due to their gene mutation, had disseminated fungal infections. One patient, who had severe disseminated coccidioidomycosis before starting ruxolitinib, acutely worsened after five months of ruxolitinib treatment, which required the cessation of ruxolitinib. Prior to ruxolitinib treatment, the patient's Th17 levels were normal, and she had very low CD4^+^, CD8^+^, and NK cells (41). The second patient experienced severe dermatophytosis to bilateral feet that was resistant to all antifungal treatments. After 4 weeks of treatment with ruxolitinib, the dermatophytosis worsened, requiring cessation of the drug (44).

In a study by Sampaio et al., 12% of patients with STAT1 GOF mutations were found to have disseminated *Coccidioides* or *Histoplasmosis*. Further investigation revealed that these mutations caused aberrant regulation of INF-γ and IL-12 signaling, leading to suppressed immune responses to disseminated mycobacteria and dimorphic yeast (5). Additionally, an *in vivo* experiment showed that neutrophils from STAT1 GOF mutation patients are highly primed for a pro-inflammatory state, characterized by cytokine release, degranulation, and transcription. Treatment with ruxolitinib in these patients had little to no effect and may even exacerbate the neutrophil pro-inflammatory state. This suggests that the SAT1 GOF neutrophil immune pathway operates through a separate pathway than ligand-induced STAT1 phosphorylation. Although endemic mycosis infections are not typically associated with a pro-inflammatory neutrophilic state, this immune pathway play a role in such infections (49). This raises that while ruxolitinib treatment can repair the immune defects causing CMC in STAT1 GOF mutations, it does not alleviate the immune defects responsible for invasive fungal diseases. The specifics of this immune pathway remain unclear.

### STAT4 LOF

In a case report of a Brazilian patient who is heterozygous for STAT4 LOF, the patient presented with paracoccidioidomycosis (50). It appears that an autosomal dominant STAT4 LOF leads to impaired IL-12-dependent IFN-γ immunity, resulting in an increased risk of paracoccidioidomycosis (36).

### JAK inhibitors fungal immunosuppression management in endemic areas

This case highlights the critical role of the JAK1 and JAK2 signaling pathways in antifungal defense, particularly through the INF-γ-JAK1-STAT axis. While ruxolitinib is effective in managing hematologic disorders, it underscores the potential to increase susceptibility to fungal infections, such as coccidioidomycosis, especially in endemic regions. As the U.S. Food and Drug Administration expands approval for JAK inhibitor treatments for numerous diseases, clinicians must be aware of the increased risk of invasive fungal infections in JAK1 inhibitors. A key takeaway is the importance of regular screening for fungal infections in patients on JAK inhibitors. This case also suggests that the increased risk of fungal infections is closely related to the immunosuppressive effects of ruxolitinib rather than the underlying hematological, autoimmune, or malignant condition.

However, limitations include the reliance on individual case reports and a lack of large-scale, randomized studies examining fungal infection risks with JAK inhibitors. Future research should focus on delineating the specific immune pathways affected by coccidioidomycosis immunity within the JAK-STAT pathway, considering whether these immune pathways increase the risk of endemic fungal infections compared to the standard exposure risk. Since these endemic fungal infections are specific to a certain geographical region, clinical trials should be conducted in these areas to better stratify actual risk. Additionally, exploration of alternative treatment strategies or more selective JAK inhibitors is needed to mitigate fungal risks. Further studies are needed to determine the efficacy and safety of antifungal prophylaxis in this patient population, balancing the risks of adverse outcomes with the benefits of infection prevention.

## Conclusion

This case demonstrates the critical role of JAK1 and JAK2 gene transcription in antifungal defense, particularly in patients receiving ruxolitinib therapy, a JAK1 and JAK2 inhibitor, for PV or myelofibrosis. The patient's relapse of pulmonary coccidioidomycosis after discontinuing itraconazole while still on ruxolitinib highlights the need for continuous monitoring and a discussion on the risks and benefits of continuing antifungal treatment. Although, identification and observations from similar cases would strengthen this clinical hypothesis. Furthermore, clinicians should regularly screen patients taking JAK inhibitors in endemic regions for coccidioidomycosis. This case underscores the crucial role JAK1 inhibition plays in antifungal defense and raises the question of whether more selective JAK inhibitors, with avoidance of JAK1 inhibition, could mitigate fungal risks while maintaining disease control. Further research is needed to clarify best practices for managing antifungal prophylaxis in patients on JAK inhibitors in endemic areas.

## Appendix

**Table A1:** Confirmatory serologic testing for coccidioidomycosis.

**Coccidioidomycosis serologic test**	**Sensitivity (%)**	**Specificity (%)**	**Notes**
Complement fixation (CF) antibody titer	70–90% (disseminated)	~90%	Rising CF titers correlate with disease activity; can remain elevated for months (8, 51)
Immunodiffusion (ID) IgM and IgG	IgM: ~75% (acute), IgG: ~80–85%	>95%	IgM appears early; IgG indicates ongoing or disseminated infection (8, 51)
EIA (enzyme immunoassay) IgG/IgM	>90% combined	~85–95%	Most sensitive early test; occasional false positives (8, 51, 52)

Sensitivity and specificity are approximate ranges based on published data: IDSA guidelines, CDC reports, and large cohort studies. Performance varies by patient population (immunosuppressed vs. immunocompetent) and disease stage.

**Table A2:** Confirmatory serologic testing for other endemic and opportunistic mycoses.

**Mycotic infection**	**Serologic test**	**Sensitivity (%)**	**Specificity (%)**	**Notes**
Histoplasmosis	Complement fixation (CF) antibody titer	70–90% (subacute/chronic)	~90%	Lower sensitivity in acute pulmonary infection (53–55)
	Immunodiffusion (ID) M and H bands	~70%	>95%	M band = acute/chronic infection; H band = active infection (53, 55, 56)
	Histoplasma antigen (urine/serum)	Urine: ~90% (disseminated AIDS)	~95%	Preferred in disseminated disease; cross-reactivity with other fungi (Blastomyces, Paracoccidioides) (53–55)
Paracoccidioidomycosis	Immunodiffusion	~80–90%	>95%	Regional variability in test performance (57)
	Complement fixation	~70–85%	~90%	Used less frequently (57)
Cryptococcosis	Serum cryptococcal antigen (CrAg, latex agglutination or lateral flow)	>95% (disseminated)	>95%	High sensitivity in HIV/AIDS; preferred test (58, 59)
Pneumocystis jirovecii	Serum Beta-D-Glucan	~90%	~80%	Not specific for Pneumocystis; positive in other fungal infections (60–62)
	PCR (BAL fluid)	>95%	~90%	Preferred for diagnosis; no serologic antibody testing available (60–62)
Aspergillosis	Serum galactomannan antigen	~70% (invasive disease)	~85–90%	False positives with certain antibiotics (e.g., piperacillin/tazobactam) (63, 64)
	Beta-D-Glucan	~80%	~80%	Non-specific; positive in multiple fungal infections (63, 64)

Sensitivity and specificity are approximate ranges based on published data: IDSA guidelines, CDC reports, and large cohort studies. Performance varies by patient population (immunosuppressed vs. immunocompetent) and disease stage.
